# Unipolar and Bipolar Plasma Electrolytic Oxidation (PEO) Coatings with Zeolite Additives for Photocatalytic Applications

**DOI:** 10.3390/molecules31101752

**Published:** 2026-05-20

**Authors:** Kristina Mojsilović, Rastko Vasilić, Marko Dević, Nenad Tadić

**Affiliations:** Faculty of Physics, University of Belgrade, Studentski trg 12-16, 11001 Belgrade, Serbia; marko.devic@ff.bg.ac.rs (M.D.); nenad.tadic@ff.bg.ac.rs (N.T.)

**Keywords:** plasma electrolytic oxidation, zeolites, bipolar, unipolar, photocatalysis

## Abstract

Plasma electrolytic oxidation (PEO) enables the fabrication of multifunctional oxide coatings with embedded active phases, offering a promising route for durable photocatalytic surfaces in water purification. This study examines how the electrical regime affects particle incorporation and photocatalytic performance. Coatings were produced under a 50% duty cycle in both unipolar mode and during the anodic part of the bipolar mode. A silicate-based electrolyte was modified with zeolites (Y and ZSM5), used in pristine form, Zn-loaded form, and combined with ZnO nanoparticles, to enhance catalytic activity. Photocatalytic performance was evaluated via methyl orange degradation under simulated solar irradiation for 6 h. The highest efficiency (~45%) was achieved with unipolar coatings containing Y zeolite and ZnO. In contrast, bipolar coatings with combined Y and ZnO showed lower efficiency (~35%). Although lower than typical powder photocatalysts, these results are notable since active phases are directly embedded in the coating, and both modes improve the photocatalytic activity by ~10% compared to the standard electrolyte. Microstructural analysis revealed that bipolar coatings were more compact, limiting access to active sites. Unipolar processing enabled better particle incorporation and a morphology more favorable for photocatalytic activity, making it the more effective regime for developing PEO-based photocatalytic coatings.

## 1. Introduction

With growing pressure on global water resources, the impacts of climate change are making water scarcity an increasingly urgent challenge. Consequently, improving wastewater treatment and preserving available water supplies have become central topics for researchers worldwide. Among the numerous approaches explored for water purification, photocatalysis has attracted considerable attention due to its ability to degrade organic contaminants effectively [1,2,3]. However, many photocatalytic materials are typically applied in powder form, which introduces an additional separation step after treatment. This post-treatment removal is both time-consuming and costly, limiting the practicality of such systems [4,5,6]. Immobilizing photocatalytically active compounds onto inexpensive and stable substrates offers a promising alternative, as it eliminates the need for particle recovery. In this context, the development of coatings with highly developed photoactive surfaces could represent an efficient strategy for the treatment of wastewaters containing organic pollutants.

To improve photocatalytic performance, considerable research attention has been devoted to the design of composite photocatalysts. Plasma electrolytic oxidation (PEO) has emerged as a suitable technique for producing robust oxide coatings capable of incorporating functional components [7,8,9]. One promising concept involves the fabrication of hybrid PEO coatings containing immobilized zeolites, where the zeolite phase can be engineered to support photocatalytic activity [10,11]. PEO is an established industrial surface modification process that transforms the surfaces of valve metals such as Al, Mg, Ti, and Zn, as well as their alloys, into ceramic-like oxide layers [12,13,14]. Numerous studies have demonstrated that particles suspended in the electrolyte can become incorporated into the growing oxide layer during PEO treatment [7,8,15,16,17,18]. The presence of such particles generally promotes the formation of thicker and more compact coatings, which can enhance wear and corrosion resistance. At the same time, particle addition may influence the dielectric breakdown behavior, as well as the chemical and phase composition of the resulting coatings, and can introduce additional functional properties including luminescent or photocatalytic activity [10,11]. A wide range of functional additives has therefore been explored in PEO electrolytes. Among them, so-called nanocontainers, such as zeolites, layered double hydroxides, and metal–organic frameworks, are of particular interest because they possess well-defined internal structures that allow them to act not only as structural fillers but also as carriers of functional ions or compounds [10,11,19,20].

Zeolites represent an especially promising class of such additives. They are crystalline aluminosilicate materials characterized by a three-dimensional framework composed of interconnected channels and cavities with molecular-scale dimensions. This porous architecture provides a large internal surface area and enables the accommodation of a variety of cations within the framework. Importantly, the extra-framework cations can be readily exchanged with other metal ions through relatively simple ion-exchange processes, making zeolites highly tunable materials in terms of chemical composition and functionality [21]. Owing to these properties, zeolites are widely used as adsorbents, ion exchangers, and heterogeneous catalysts in a wide range of industrial applications.

Beyond these traditional roles, zeolites have also attracted attention in photocatalytic systems. Their porous framework can serve as a host matrix for semiconductor nanoparticles, preventing particle agglomeration and improving the dispersion of photoactive phases [22,23]. In addition, the presence of the zeolite structure may enhance the adsorption of organic molecules near photocatalytically active sites, thereby increasing the efficiency of photocatalytic reactions [22,23]. In some cases, the zeolite framework itself can participate in photoactivation processes or facilitate charge separation when coupled with semiconductor oxides [10,11].

Previous investigations have demonstrated that zeolites can be successfully incorporated into oxide coatings during PEO processing. In particular, coatings containing Ce-loaded zeolites have shown notable photocatalytic performance, while simultaneously improving resistance to degradation in aggressive environments [10,11,24]. Nevertheless, the mechanism by which zeolites become incorporated, whether through inert embedding or reactive participation in the coating growth, has remained unclear. Some evidence suggests that inert incorporation occurs under so-called soft sparking conditions during the PEO process [25,26]. In the present work, two types of zeolites, ZSM5 and Y, are instead loaded with Zn species. This choice is motivated by the well-known photocatalytic properties of ZnO and by previous reports confirming that Zn-based compounds can be successfully incorporated into PEO coatings [27,28,29].

The primary objective of this study is to compare unipolar and bipolar electrical regimes during PEO processing in order to determine which approach is more effective in producing coatings with sufficiently developed active surfaces for photocatalytic applications. More specifically, zeolites are employed as supports for photoactive species, potentially simplifying photocatalyst preparation while enhancing the efficiency of photocatalytic degradation of a model organic pollutant. To this end, photocatalytic coatings are fabricated by PEO treatment in electrolytes containing the selected zeolites with or without ZnO nanoparticles. The resulting coatings are then characterized in terms of their surface morphology, chemical and phase composition, and their potential performance as photocatalysts in wastewater treatment applications.

## 2. Results

The designation of samples and the corresponding electrolyte compositions are summarized in Table 1. For completeness, the table also includes the measured pH and electrical conductivity of the electrolytes, as these parameters are known to influence the PEO process and are relevant for the interpretation of the results discussed below. It should be noted that the pH and electrical conductivity values of the utilized electrolytes have not changed significantly after the PEO processing.

### 2.1. Voltage Evolution

**Figure 1:**
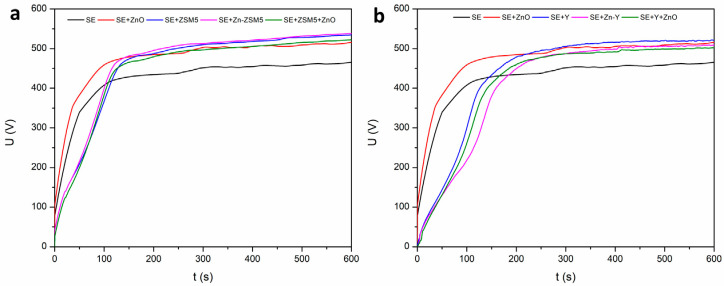
Voltage evolution in unipolar electrical regime: (**a**) with ZSM5 zeolite; (**b**) with Y zeolite.

In the unipolar electrical regime, the voltage–time response follows the classical behavior typically observed for PEO, as illustrated in Figure 1. At the beginning of the treatment, the applied voltage increases almost linearly with processing time. This initial stage corresponds to conventional anodization, during which a compact barrier-type oxide layer forms at the metal–electrolyte interface. The oxide film grows uniformly under the influence of a strong electric field, with ionic migration of Al^3+^ cations outward and O^2−^/OH^−^ species inward through the developing oxide layer. As the film progressively thickens, its electrical resistance and dielectric strength increase, resulting in a continuous rise in the applied voltage required to maintain the imposed current density. Once the local electric field across the oxide exceeds the dielectric breakdown threshold, localized dielectric breakdown occurs. This event marks the transition into the characteristic plasma stage of the PEO process and is manifested by the appearance of numerous microdischarges distributed across the aluminum surface. The microdischarges locally melt and partially ionize both oxide and electrolyte species, followed by rapid quenching and re-solidification. As a result, the initially compact oxide layer evolves into a thicker ceramic coating with a characteristic porous morphology composed of re-solidified oxide and incorporated electrolyte-derived species.

Under unipolar operation, the addition of solid particles to the electrolyte, namely ZnO and different zeolite types, noticeably affects the voltage evolution during processing. In all cases where particles were introduced, the breakdown voltage increased compared to the particle-free electrolyte. This indicates that the presence of suspended particles modifies the dielectric properties and growth kinetics of the initially formed oxide layer. In particular, the onset of microdischarges was delayed in electrolytes containing zeolite particles, with the breakdown event occurring approximately 20 s later than in the standard electrolyte. Such a delay suggests that particle incorporation and/or particle adsorption at the oxide–electrolyte interface alters the local electric field distribution and increases the effective dielectric strength of the growing oxide film.

Following breakdown, the voltage measured throughout the remainder of the treatment remains consistently higher in electrolytes containing particles than in the standard electrolyte. This behavior can partly be attributed to modifications in the electrolyte properties, including slight changes in conductivity (Table 1) due to particle suspension and possible release of ionic species from particle surfaces [16,30]. In addition, the physical presence of particles in the discharge channels and near the coating surface may influence discharge dynamics by affecting plasma stability, heat dissipation, and local electric field concentration [31]. Particles can also become incorporated into the growing oxide layer through electrophoretic transport or entrapment within discharge channels, which may further modify the electrical resistance and growth behavior of the coating [30,32].

**Figure 2:**
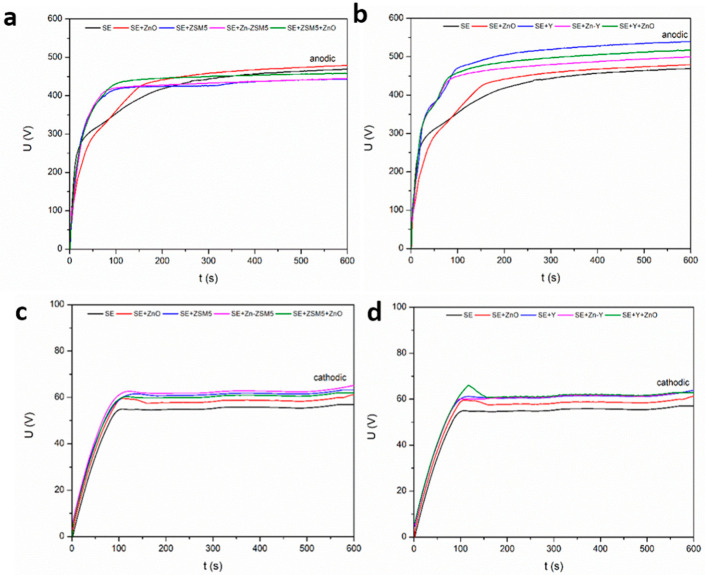
Voltage evolution in bipolar electrical regime: (**a**) the evolution of the anodic part with ZSM5 zeolite; (**b**) the evolution of the anodic part with Y zeolite; (**c**) the evolution of the cathodic part with ZSM5 zeolite; (**d**) the evolution of the cathodic part with Y zeolite.

When the bipolar electrical regime is applied, the temporal evolution of the voltage differs significantly from that observed under unipolar operation (Figure 2). In the present study, the ratio of cathodic to anodic current densities was fixed at (R = 1.3), a condition previously reported to promote the so-called “soft sparking” regime [31,32,33,34,35,36,37,38]. In this regime, the discharge activity becomes more stable and less energetic compared to conventional PEO, which often results in denser coatings with improved structural integrity. However, in the present experiments the characteristic transition to soft sparking was not reached within the applied processing time of 10 min, indicating that the coating system remained within the conventional discharge regime throughout the treatment.

The evolution of the cathodic voltage (Figure 2c,d) during bipolar processing is closely related to the progressive development of the oxide layer, including increases in thickness, reductions in defect density, and improvements in dielectric strength [31,32]. The application of cathodic pulses plays a key role in accelerating these structural changes. Cathodic polarization can facilitate the removal of weakly bonded surface species and loosely attached oxide fragments generated during discharge events. At the same time, cathodic pulses may promote field-assisted rearrangement and densification of the oxide structure, as well as enhanced charge accumulation within subsurface regions of the coating. These processes contribute to a gradual reduction in structural defects and discharge intensity. As a result, the breakdown threshold increases and the discharge behavior becomes more stable, which ultimately leads to improved dielectric integrity and a more compact coating microstructure [31,32,33,34,35,36,37,38].

As in the unipolar regime, the influence of particle additions is also evident under bipolar operation. In particular, the breakdown voltage is again increased in electrolytes containing zeolite particles, indicating that the presence of these particles modifies the early-stage dielectric properties of the oxide layer and the conditions required for discharge initiation. On the other hand, the presence of ZnO alters the voltage evolution only slightly.

### 2.2. Morphology and Chemical and Phase Compositions of PEO Coatings

**Figure 3:**
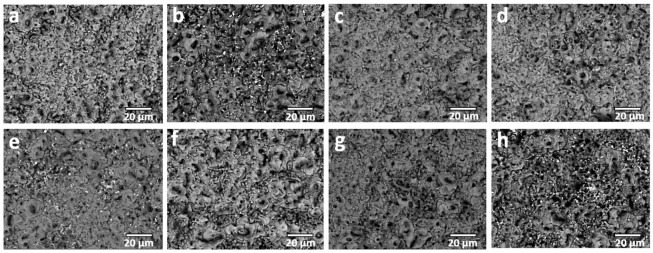
Surface SEM micrographs for samples treated in unipolar processing mode: (**a**) SE; (**b**) SE + ZnO; (**c**) SE + ZSM5; (**d**) SE + Zn-ZSM5; (**e**) SE + ZSM5 + ZnO; (**f**) SE + Y; (**g**) SE + Zn-Y; (**h**) SE + Y + ZnO.

The surface morphology of the coatings was systematically examined by SEM analysis, as presented in Figure 3. In agreement with the well-established characteristics of PEO processing conducted under a unipolar electrical regime, all treated specimens display a highly developed and heterogeneous surface morphology. This morphology is predominantly characterized by a high density of pores, so-called “pancake” features, and fine crater structures [12,13,14]. Notably, these morphological signatures are consistently observed across all investigated samples, irrespective of the specific type of zeolite incorporated into the electrolyte formulation. The formation of such features can be attributed to the intense and localized microdischarge events inherent to the unipolar PEO regime. These transient plasma discharges induce rapid localized melting of the substrate surface, followed by extremely fast solidification upon contact with the surrounding electrolyte [12,13,14]. This repetitive sequence of melting and quenching results in the development of a porous, cratered architecture with resolidified splats, giving rise to the characteristic “pancake” morphology.

**Figure 4:**
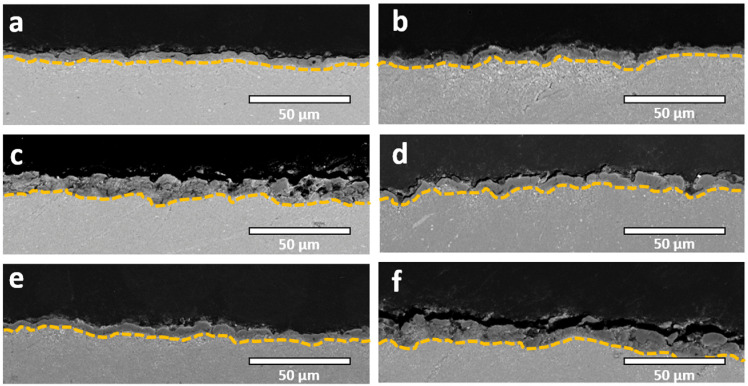
Cross-sectional SEM micrographs for samples treated in unipolar processing mode: (**a**) SE; (**b**) SE + ZnO; (**c**) SE + ZSM5; (**d**) SE + Y; (**e**) SE + Zn-Y; (**f**) SE + Y + ZnO.

Cross-sectional analysis (Figure 4) further reveals that the coatings are relatively thin yet exhibit a notable degree of compactness. In contrast, the incorporation of zeolite particles promotes the formation of a more pronounced layered structure, consisting of a well-developed, highly porous outer layer superimposed on a dense, adherent inner barrier layer. Additionally, a measurable increase in coating thickness is observed upon the addition of ZnO and zeolites, with the maximum thickness achieved when both additives are simultaneously present in the electrolyte (Table 2). This suggests a synergistic effect between ZnO particles and zeolitic phases in facilitating coating growth.

In comparison to coatings produced in the standard electrolyte, a slight reduction in surface roughness is observed when particulate additives are introduced, whereas the overall porosity tends to increase (Table 3). This trend may appear counterintuitive, as particulate incorporation is commonly associated with partial pore sealing and an increase in surface roughness [30]. However, this behavior may be explained by considering the altered electrical and thermal conditions during processing. Specifically, the presence of ZnO and zeolites leads to higher operating voltages and more energetic microdischarge activity (Figure 1), likely due to modified electrolyte conductivity, localized electric field increase, and changes in heat dissipation dynamics [32,39]. These conditions promote the formation of larger and more energetic discharge channels, which in turn generate increased porosity and enlarged surface features rather than pore closure.

This interpretation is further supported by quantitative pore size analysis. The average pore diameter for coatings formed in the standard electrolyte is approximately 1.55 µm, whereas samples processed with particulate additives exhibit significantly larger pore sizes, ranging from 2.54 µm up to 6.25 µm. Such an increase in pore dimension is consistent with intensified discharge behavior and supports the hypothesis that additive-induced modifications to the discharge regime play a dominant role in governing the resulting surface morphology.

**Figure 5:**
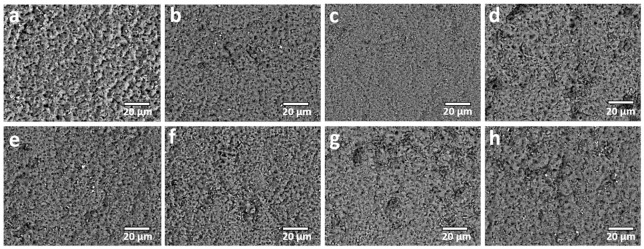
Surface SEM micrographs for samples treated in bipolar processing mode: (**a**) SE; (**b**) SE + ZnO; (**c**) SE + ZSM5; (**d**) SE + Zn-ZSM5; (**e**) SE + ZSM5 + ZnO; (**f**) SE + Y; (**g**) SE + Zn-Y; (**h**) SE + Y + ZnO.

The porosity of coatings formed under bipolar electrical conditions is significantly reduced compared to that of coatings produced in analogous unipolar regimes (Table 3), indicating a fundamental alteration in the discharge behavior and oxide growth dynamics [40]. This decrease in porosity is consistent with the observed modifications in surface morphology, where the characteristic pore diameters are confined to a relatively narrow range of approximately 0.5–1.1 µm. In contrast to the unipolar regime, which typically yields a highly cratered and irregular surface topography associated with intense, localized microdischarge activity, the bipolar regime promotes the formation of a comparatively smoother and more homogeneous surface (Figure 5). This morphological transition can be directly attributed to the periodic reversal of polarity, which fundamentally modifies the plasma–electrochemical interactions at the oxide–electrolyte interface.

More specifically, the alternating anodic and cathodic pulses inherent to the bipolar regime inhibit the sustained localization of discharge channels that is characteristic of unipolar processing. In unipolar conditions, persistent anodic polarization facilitates continuous oxide growth at fixed discharge sites, leading to the development of large discharge channels and pronounced pore structures. By contrast, in the bipolar regime, the cyclic polarity inversion disrupts this steady-state growth, periodically quenching active discharge sites and redistributing energy input across the surface. This results in a more uniform spatial distribution of microdischarges, as well as enhanced lateral spreading and re-solidification of molten oxide. Consequently, the tendency towards localized material accumulation around discharge channels is diminished, promoting the formation of a denser, less porous oxide layer with improved structural uniformity [41,42,43]. In parallel, this more homogeneous discharge behavior is expected to reduce surface roughness, as the suppression of large, energetically dominant discharge events limits the formation of deep craters and protrusions.

**Figure 6:**
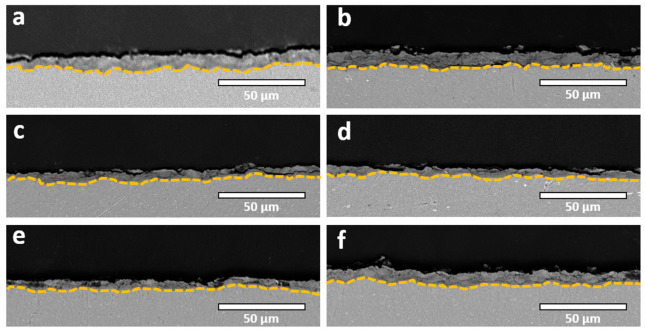
Cross-sectional SEM micrographs for samples treated in bipolar processing mode: (**a**) SE; (**b**) SE + ZnO; (**c**) SE + ZSM5; (**d**) SE + Y; (**e**) SE + Zn-ZSM5; (**f**) SE + ZSM5 + ZnO.

Despite these advantages in terms of morphology and porosity, the coatings produced in the present study remain relatively thin. This is primarily due to the fact that the processing duration was insufficient to reach the so-called soft sparking regime, which is typically associated with a transition to lower-energy, more uniformly distributed discharges that favor accelerated coating thickening [31,32,33,34,35,36,37,38]. Cross-sectional observations (Figure 6) confirm the limited thickness of the oxide layers, with an additional reduction observed in electrolytes containing zeolite additives. The incorporation of zeolitic particles appears to further suppress coating growth, likely through modifications of the discharge characteristics and local electric field distribution. On the other hand, in the case of ZnO-containing electrolytes, where the coating thickness is maximized, this behavior correlates well with the voltage–time response (Figure 2), which closely resembles that of the standard electrolyte, suggesting that the underlying discharge mechanisms and oxide growth kinetics remain largely unaltered in this system.

The EDS analysis indicates that all coatings are predominantly composed of aluminum oxide, for both unipolar and bipolar processing modes. An increase in silicon content is consistently observed with the addition of zeolites to the electrolyte, in both electrical regimes, confirming the incorporation of silicate-based species originating from the zeolitic structure. However, a comparative assessment of elemental concentrations suggests that particle incorporation is more effective under unipolar conditions (Table 4 and Table 5). This is evidenced by the higher weight percentages of Si (from zeolites) and Zn (from ZnO or Zn-exchanged zeolites) detected in coatings produced in the unipolar mode.

A notable observation is that the simultaneous addition of zeolite Y and ZnO results in the highest measured Zn content, exceeding that obtained with ZnO alone or in combination with ZSM5. This trend implies that zeolite Y facilitates more efficient Zn incorporation into the coating. A plausible explanation lies in its physicochemical characteristics, such as larger pore size, higher ion-exchange capacity, and greater accessibility of active sites compared to ZSM5 [44]. These properties likely enhance its ability to act as a carrier or “vehicle” for Zn species, promoting their transport toward and subsequent incorporation into the growing oxide layer during the PEO process.

**Figure 7:**
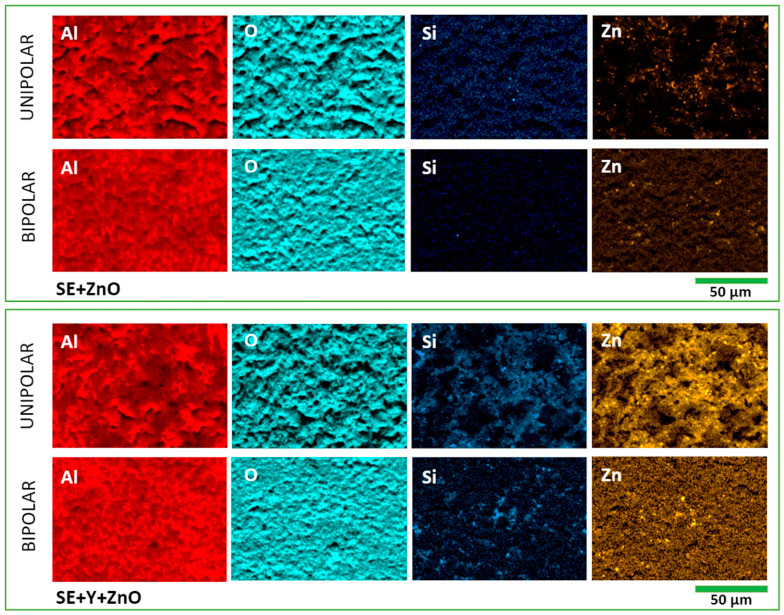
EDS mapping of samples’ surfaces treated in electrolytes containing ZnO and ZnO combined with Y zeolite, for unipolar and bipolar processing conditions.

EDS elemental mapping further supports these findings by revealing distinct differences in particle distribution (Figure 7). In electrolytes containing only ZnO, localized agglomeration is observed, indicating limited dispersion and, consequently, spatially heterogeneous incorporation. In contrast, when ZnO is introduced together with zeolites, the Zn signal appears more uniformly distributed across the coating surface [45]. This improved dispersion suggests that zeolites contribute to stabilizing Zn-containing species in the electrolyte and facilitate their more homogeneous incorporation during PEO. As a result, the potential functional influence of Zn (e.g., on photocatalytic properties) is expected to be more uniformly expressed across the entire surface, rather than being confined to isolated regions. This effect is particularly relevant in the bipolar regime, where the more uniformly distributed microdischarges may support spatially homogeneous incorporation pathways. Nevertheless, despite this apparent advantage in distribution, the overall incorporation efficiency, quantified by the total Zn and Si content, remains higher in coatings formed under unipolar conditions. This suggests that while bipolar processing may enhance dispersion through its discharge characteristics, unipolar processing provides more favorable conditions for the net uptake and retention of particulate or ion-derived species within the coating matrix.

**Figure 8:**
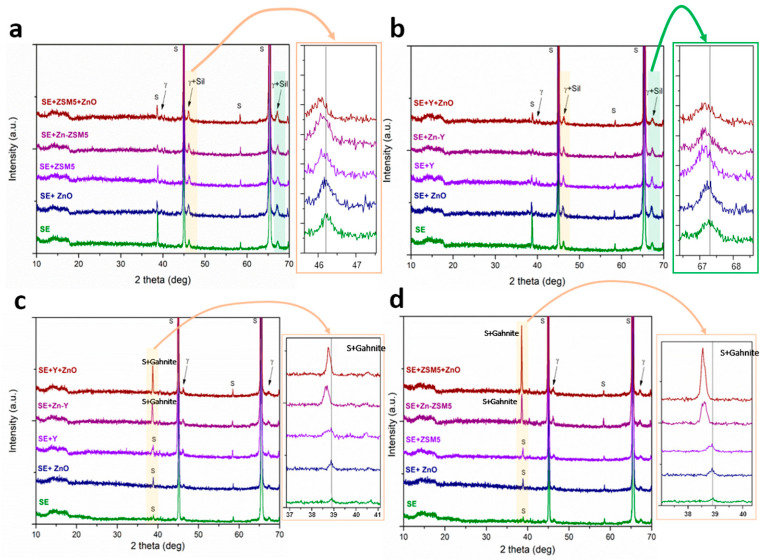
X-ray diffraction patterns for samples treated in unipolar (**a**,**b**) and bipolar (**c**,**d**) processing modes with high-resolution patterns in the section of interest. S represents substrate reflections (Al), γ is γ-Al_2_O_3_, γ + Sil represents superpositioned reflections of γ-Al_2_O_3_ and sillimanite, and S + Gahnite represents superpositioned reflections of the substrate and gahnite.

X-ray diffraction patterns of PEO coatings formed on Al substrates in electrolytes containing ZSM5 and Y-type zeolites under unipolar processing conditions are presented in Figure 8a,b. The diffractograms are dominated by reflections originating from the aluminum substrate (PDF 00–001-1176) (denoted by S) and gamma alumina γ-Al_2_O_3_ (JCPDS 10–0425). Notably, diffraction maxima corresponding to either the parent zeolites or their Zn-exchanged counterparts are absent. This absence may, in principle, arise from (i) low zeolite content and high dispersion, and/or (ii) partial amorphization under the extreme thermal conditions of microdischarges. However, the XRD data provide additional evidence that the zeolites do not merely amorphize but actively participate in phase formation [10].

Specifically, upon incorporation of zeolites into the electrolyte, additional broad diffraction features emerge, denoted by γ + Sil, corresponding to a combination of γ-Al_2_O_3_ and sillimanite (JCPDS 22–0018). A more detailed examination reveals that, in coatings formed without zeolite addition, the diffraction maxima located at approximately 2θ ≈ 46.2° and 67.3° can be assigned predominantly to crystalline γ-Al_2_O_3_. In contrast, when zeolites are present, these peaks exhibit a discernible shift and broadening toward positions characteristic of sillimanite-type aluminosilicates. This behavior strongly suggests that the original γ-Al_2_O_3_ phase partially reacts with silicate species originating from the electrolyte and/or decomposed zeolite structures, leading to the in situ formation of mixed aluminosilicate phases. The resulting broad diffraction envelopes indicate a coexistence of poorly crystallized γ-Al_2_O_3_ and sillimanite, likely reflecting a non-equilibrium, rapidly solidified microstructure with limited long-range order [10]. This systematic shift is absent in coatings formed without zeolites and therefore cannot be attributed solely to changes in γ-Al_2_O_3_ crystallinity. Instead, it indicates the formation of mixed Al–Si phases. The incorporation mechanism of zeolites in the unipolar regime can therefore be interpreted as a combination of physical entrapment of zeolitic species and chemical transformation through plasma-assisted reactions with molten alumina, i.e., partially inert incorporation. The locally high temperatures within microdischarges facilitate the breakdown of the zeolite framework, releasing SiO_4_ and AlO_4_ units that subsequently participate in the formation of secondary aluminosilicate phases. Consequently, while the original zeolite structure does not survive the PEO process in its crystalline form, the observed γ-Al_2_O_3_ to sillimanite transition provides indirect but clear evidence of reactive incorporation, rather than purely inert entrapment or complete amorphization.

In the case of the bipolar processing mode (Figure 8c,d), the XRD patterns reveal the coexistence of substrate-derived aluminum reflections (S) and pronounced peaks corresponding to gahnite (ZnAl_2_O_4_, PDF 96-900-7046) when Zn-species and zeolites appear together in the PEO electrolyte. The formation of this phase indicates the development of a Zn-Al-O system, significantly different from the predominantly Al-O-Si chemistry observed in the unipolar regime, as it represents a spinel structure, which could be beneficial for application in photocatalysis [46,47].

During the anodic half-cycle, intense microdischarges generate transient high-temperature regions where aluminum from the substrate is oxidized to Al_2_O_3_, and Zn-species are thermally activated [32]. Under such conditions, solid-state or molten-phase reactions between ZnO (or Zn-containing intermediates) and Al_2_O_3_ become thermodynamically favorable, leading to the formation of the spinel ZnAl_2_O_4_ (gahnite). The repeated alternation between anodic oxidation and cathodic modification cycles enhances mixing at the microscopic level, effectively promoting the nucleation and growth of this mixed-oxide phase. Similarly to the γ-Al_2_O_3_ and sillimanite mixing in unipolar mode, gahnite is found superpositioned onto the Al reflections for coatings made in bipolar mode.

An additional contributing factor in bipolar mode is the increased discharge density and more uniform spatial distribution of plasma events, which can lead to improved homogenization of incorporated species. Compared to unipolar processing, this results in a more efficient incorporation of electrolyte-derived cations and a higher probability of forming thermodynamically stable mixed oxides rather than retaining separate or weakly interacting phases.

In both unipolar and bipolar processing modes, an amorphous phase contribution can be noticed in the region between 2θ values of 10° and 20°, which is typical for silicate-based electrolytes and is consistent with rapid solidification during microdischarge events [31,32].

**Figure 9:**
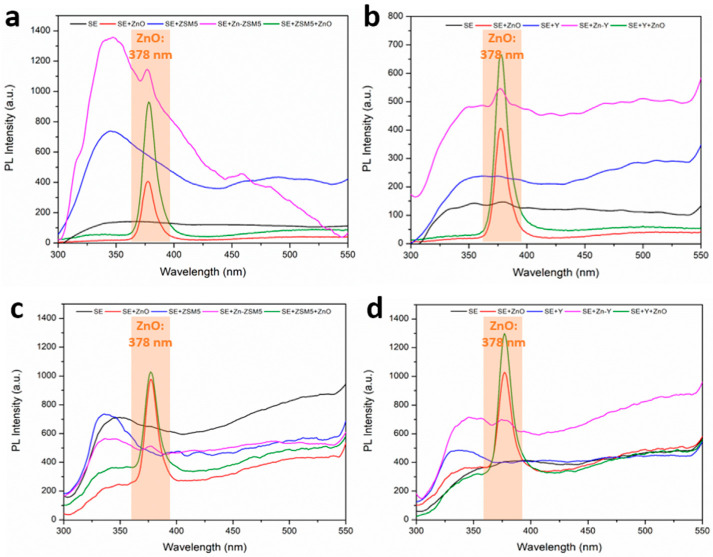
PL spectra (excitation wavelength λex = 285 nm) of the samples treated in unipolar processing mode: (**a**) ZSM5-related additives; (**b**) Y-related additives; and bipolar processing mode: (**c**) ZSM5-related additives; (**d**) Y-related additives.

To interpret the potential role of zeolitic additives in promoting the incorporation of Zn-containing species, and particularly ZnO, a well-established photocatalytically active phase [48], PL spectroscopy was employed. In the unipolar regime (Figure 9a,b), the PL spectra of coatings formed in electrolytes containing zeolites exhibit a pronounced and broad emission spanning approximately 300–600 nm. This emission is characteristic of Al_2_O_3_ and is commonly attributed to intrinsic defect centers, predominantly oxygen vacancies in various charge states (F and F^+^ centers). These defects are well-known to act as radiative recombination centers in alumina-based oxide films. More specifically, emission bands centered around 327 nm and 693 nm can be assigned to F^+^ centers in Al_2_O_3_, consistent with previous reports [49,50]. The prominence of these bands indicates a high density of defect states within the oxide matrix, which is typical for PEO coatings due to the rapid solidification and non-equilibrium conditions inherent to the process.

Upon introduction of Zn-species into the electrolyte, either directly as ZnO particles or indirectly via Zn-exchanged zeolites, an additional emission feature emerges at approximately 378 nm, which is characteristic of ZnO. Notably, the intensity of this ZnO-related signal is significantly boosted when ZnO is co-introduced with zeolites, suggesting that zeolitic structures facilitate the transport and incorporation of Zn species into the growing oxide layer. This observation supports the hypothesis that zeolites act as effective carriers or reservoirs of Zn ions, enabling their sustained release and subsequent incorporation during microdischarge events.

A similar but more pronounced behavior is observed under bipolar processing conditions (Figure 9c,d). The ZnO-related emission at around 378 nm becomes even more intense. Consistent with the unipolar case, the presence of zeolites further amplifies this signal. Importantly, even in systems containing only Zn-exchanged zeolites (without added ZnO powder), a detectable ZnO-related emission is observed, reinforcing the role of zeolites as active Zn sources during the PEO process.

It should be noted that although excitation at 285 nm lies within the ZnO absorption region, as confirmed by the optical bandgap of ~385 nm determined for a comparable coating [27], Zn-containing species represent only a minor fraction of the predominantly alumina-based coatings (Table 4 and Table 5). Moreover, the consistent observation of the ~378 nm ZnO near-band-edge emission across all samples confirms that excitation light reaches ZnO crystallites without full attenuation. Therefore, strong self-absorption or inner filter effects are not expected to significantly distort the relative PL intensities measured under the employed experimental conditions [51].

### 2.3. Photocatalytic Properties of Formed PEO Coatings

**Figure 10:**
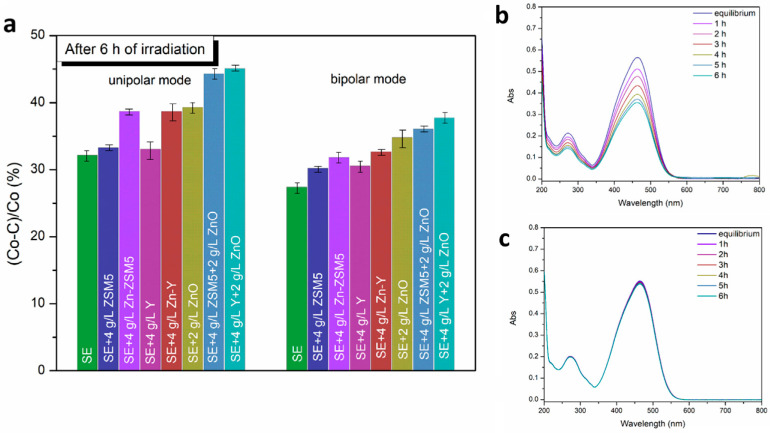
(**a**) Efficiency of photocatalytic degradation of MO on oxide layers formed in unipolar and bipolar processing mode (the error represents a standard deviation from four measurements); (**b**) Absorbance spectra of MO after up to 6 h of irradiation in the presence of SE + ZSM5 sample obtained in unipolar mode; (**c**) Absorbance spectra of MO after 6 h in the darkness in the presence of SE + Y + ZnO sample treated in bipolar mode.

The photocatalytic performance of all PEO coatings produced under both unipolar and bipolar regimes after 6 h of irradiation using a solar spectrum simulating lamp is summarized in Figure 10a. In this analysis, C_0_ represents the initial concentration of methyl orange (MO), while C corresponds to its concentration at a given irradiation time, t. MO was selected as a model compound due to its well-established classification as a persistent environmental pollutant and its frequent use in photocatalytic degradation studies [52].

To distinguish true photocatalytic activity from adsorption phenomena, control adsorption experiments were performed in the absence of illumination. These tests involved monitoring changes in MO concentration in dark conditions. A representative example, shown in Figure 10c, demonstrates that the decrease in MO concentration due to adsorption alone is minimal. This confirms that the observed reduction in dye concentration under illumination predominantly originates from photocatalytic reactions rather than surface adsorption. Consequently, the enhanced degradation efficiency can be attributed to the presence of photocatalytically active phases incorporated in the oxide coatings.

A clear enhancement in photocatalytic activity is observed for coatings produced in electrolytes containing functional additives compared to those formed in the standard electrolyte alone, regardless of whether unipolar or bipolar electrical modes were applied (Figure 10a,b). This improvement is primarily attributed to the incorporation of photocatalytically active species (e.g., ZnO), rather than to morphological changes alone.

At the same time, the results reveal systematically higher photocatalytic performance for coatings formed in the unipolar regime compared to their bipolar counterparts. This difference can be primarily linked to modifications in coating morphology, specifically the development of a more porous and structurally complex surface, which increases the available active surface area for photocatalytic reactions (as indicated in Table 3). Such morphological features facilitate improved light harvesting, reactant diffusion, and accessibility of catalytically active sites [10,11].

To further investigate the photocatalytic activity of the obtained PEO coatings, the decolorization rate constants of MO were determined using the Langmuir–Hinshelwood kinetics. The degradation rate for a pseudo-first-order reaction is described as [53]

(1) $$ln\left(\frac{C}{C_{0}}\right)=-k_{app}\times t$$

where *k_app_* is the apparent rate constant (Table 6). The fitting correlation coefficients (R^2^) for the kinetic curves of reactions for the formed PEO coatings vary from 0.95 to 0.99, which confirms that the MO waste followed a fundamentally pseudo-first-order kinetic model.

## 3. Discussion

The incorporation of zeolitic particles into the electrolyte significantly alters the PEO process, as reflected in the voltage–time response. Interestingly, the deviation in voltage evolution from the behavior observed in the standard electrolyte is considerably more pronounced when Y-type zeolite is added compared with ZSM5, although both materials belong to the zeolite family. This difference can likely be attributed to their distinct framework structures, chemical compositions, and ion-exchange characteristics. Y zeolite (FAU framework) possesses a relatively low Si/Al ratio and therefore contains a higher density of framework aluminum atoms, which results in a large number of negatively charged lattice sites that must be balanced by exchangeable cations [44,54]. Consequently, Y zeolite exhibits a high cation-exchange capacity and strong interaction with electrolyte ions. In contrast, ZSM5 (MFI framework) typically has a significantly higher Si/Al ratio, which reduces the density of exchangeable sites and makes its framework more hydrophobic and chemically inert [44,55].

Because of these differences, Y zeolite particles are more likely to interact with ionic species in the electrolyte and modify the local ionic environment near the oxide–electrolyte interface. Such interactions can influence the structure of the electrical double layer and the mobility of charge carriers involved in oxide growth. In addition, the larger pore openings and higher internal surface area of the FAU framework may facilitate adsorption of electrolyte species, which can further alter the local electric field distribution at the coating surface [44]. These effects may increase the effective dielectric strength of the growing oxide layer, thereby delaying the onset of dielectric breakdown and resulting in higher breakdown voltages. Furthermore, once microdischarges are established, particle incorporation into discharge channels and the re-solidifying oxide matrix may further amplify these differences by locally modifying the electrical and thermal properties of the coating [13].

As mentioned above, the deviation from the baseline voltage response is substantially more pronounced for electrolytes containing Y-type zeolite compared to those with ZSM-5, which is likely responsible for the observed increase in dielectric strength of the oxide layer in Y-containing electrolytes, i.e., the delayed onset of dielectric breakdown and the higher breakdown voltages observed. From a microstructural perspective, a higher breakdown voltage generally corresponds to the initiation of fewer but more energetic discharge events. This has several consequences: on the one hand, the delayed breakdown suppresses early-stage discharge activity, limiting initial pore formation and contributing to lower overall porosity (Table 3); on the other hand, once the breakdown occurs, the higher energy of individual discharges may promote localized melting and densification, further reducing pore connectivity and refining pore size distribution. Simultaneously, the reduced frequency of discharge events can limit vertical oxide growth, contributing to the observed decrease in coating thickness (Table 2).

In addition to influencing the electrical and microstructural characteristics of the coatings, the incorporation of Zn-containing species leads to the formation of ZnO within the oxide matrix, as confirmed by PL analysis. For comparison, the PL spectrum of ZnO in powder form typically consists of two features: a relatively weak near-ultraviolet emission band centered around 380 nm, attributed to the radiative recombination of free excitons, and a broad green emission band centered near 510 nm, which is generally associated with intrinsic defects such as oxygen vacancies or zinc vacancies. The relative intensities of these bands are widely used as indicators of crystalline quality and defect density in ZnO [56]. In this context, the obtained PL spectra (in both electrical regimes) reveal a dominant and relatively sharp ultraviolet emission at 378 nm, accompanied by a significantly suppressed and broadened green emission around 510 nm. This spectral signature suggests that the incorporated ZnO phase exhibits comparatively high crystallinity and a reduced concentration of defect states. Such behavior is in line with literature reports indicating that improved crystalline quality, manifested by reduced density of oxygen vacancies, zinc interstitials, and impurity-related defects, leads to enhanced near-band-edge emission and quenching of green luminescence [57]. A plausible explanation for this phenomenon can be drawn from the interaction between ZnO and the surrounding Al_2_O_3_ matrix. Previous studies have demonstrated that Al_2_O_3_ buffer layers can significantly modify the defect chemistry of ZnO films by acting as a source of oxygen [57]. During the growth, oxygen atoms can diffuse from the alumina phase into the ZnO lattice, effectively filling oxygen vacancies and thereby reducing the density of deep-level defect states. This process results in suppression of the green emission band and a concomitant enhancement of ultraviolet emission due to improved free exciton recombination. The present results strongly suggest that a similar mechanism operates in PEO coatings: the Al_2_O_3_-rich matrix formed during oxidation provides an oxygen reservoir that promotes defect healing in the incorporated ZnO phase. Furthermore, the presence of zeolites may enhance this effect by enabling a more homogeneous and controlled incorporation of Zn species, thereby facilitating the formation of better-crystallized ZnO domains within the oxide layer.

The functional implications of these structural and compositional features are clearly reflected in the photocatalytic performance of the coatings. When comparing the two electrical regimes, coatings produced under unipolar conditions usually exhibit superior photocatalytic performance. This behavior is consistent with their higher degree of porosity and more developed surface architecture, which together promote more efficient interaction between the photocatalyst and the target pollutant. The photocatalytic effect is likely governed by a combination of defect-mediated processes and adsorption-assisted degradation [10,11]. In addition to morphological factors, compositional differences also play a significant role. Surface analysis revealed a higher incorporation of zinc in unipolar coatings, predominantly present as ZnO (Table 4 and Table 5), a well-known semiconductor with strong photocatalytic properties [56].

The photocatalytic activity of ZnO is governed by its ability to generate electron–hole pairs upon light irradiation. When exposed to photons with sufficient energy, electrons (e^−^) are excited from the valence band to the conduction band, leaving behind positively charged holes (h^+^) in the valence band. These charge carriers participate in redox reactions at the surface of the material. Specifically, conduction band electrons reduce molecular oxygen to form superoxide radicals (^•^O_2_^−^), while valence band holes oxidize water or hydroxide ions to generate hydroxyl radicals (^•^OH). These highly reactive species are responsible for the oxidative degradation of organic molecules such as MO, ultimately leading to their mineralization. The overall photocatalytic degradation pathway of MO mediated by ZnO can be described through a sequence of reactions (Equations (2)–(4)) [58]:

ZnO + hν → ZnO (h^+^ + e^−^), (2)

O_2_ + e^−^ → ^•^O_2_^−^, (3)

H_2_O + h^+^ → ^•^OH + H^+^, (4)

or in a general, stoichiometrically simplified manner [58]:

h^+^ + ^•^O_2_^−^ + ^•^OH + MO → CO_2_ + H_2_O. (5)

Importantly, the combined effect between ZnO and zeolitic phases is evident in both electrical regimes. The highest levels of photocatalytic efficiency are consistently achieved in coatings where both components are present, highlighting the beneficial role of zeolite incorporation. Zeolites contribute not only by increasing surface area but also by enhancing adsorption of pollutant molecules and facilitating their proximity to active photocatalytic sites. Among the investigated systems, coatings containing Y-type zeolite exhibit the most pronounced photocatalytic activity. This superior performance can be correlated with the distinct morphological characteristics associated with this zeolite, as discussed in Section 2.1 and Section 2.2, including its influence on pore structure, particle distribution, and overall coating architecture. The highest photocatalytic activity reached approximately 45% after 6 h of irradiation. While this value is lower than those typically reported for dispersed photocatalyst powders, where degradation efficiencies often reach 100% under comparable conditions [59], it is consistent with literature data for immobilized photocatalytic systems, which generally exhibit reduced activity due to limited active surface area, mass transfer constraints, and increased charge recombination [10,11,19].

It should also be noted that a direct numerical comparison with many literature studies is complicated by the widespread use of methylene blue (MB) as a model pollutant, which is known to undergo partial self-sensitized photodegradation under visible light irradiation even in the absence of a catalyst. Studies comparing MB and MO degradation under the same conditions have consistently shown that MB degrades significantly faster, with photocatalytic rates for MB being up to 8–13 times higher than for methyl orange using equivalent TiO_2_ thin film coatings [60,61]. Furthermore, when the same photocatalytic system is tested against both dyes simultaneously, efficiencies as high as 81% are reported for MB but only ~26% for MO under identical irradiation conditions [60]. MO is an anionic azo dye classified as highly recalcitrant and refractory, with well-documented resistance to natural degradation and significant ecotoxicological concern, making it a more demanding and environmentally relevant benchmark [62].

In this context, the ~45% degradation of MO achieved by the present PEO-based coatings is directly comparable to the performance of bare, undoped TiO_2_ PEO coatings reported in the literature, which have been shown to achieve approximately 47% degradation under comparable conditions [63]. Higher efficiencies reported for PEO systems, such as 95% MB degradation, have been achieved only through transition metal doping (e.g., W-doped TiO_2_) [64], which suppresses charge recombination and extends visible light absorption, while heterojunction PEO coatings with hydrothermal post-treatment have reached up to 75% MB degradation under visible light [65].

The obtained efficiency can therefore be considered competitive for PEO-based coatings produced without dopants or post-treatment modifications, and the use of MO as the test pollutant. Nevertheless, the results also indicate significant potential for further optimization, particularly in terms of increasing active phase exposure, introducing appropriate dopants or heterojunctions, and improving charge separation.

## 4. Materials and Methods

### 4.1. Zeolite Preparation

The parent materials employed in this study were commercially available synthetic zeolites, namely MFI-type ZSM5 zeolite (H-form, Si/Al = 15) and FAU-type Y zeolite (H-form, Si/Al = 2.6), both supplied by Zeolyst International (Ghent, Belgium). Zinc incorporation was performed via a combined ion-exchange/impregnation approach. In a typical procedure, 1 g of each zeolite was dispersed in 200 mL of an aqueous 0.002 M Zn(NO_3_)_2_·6H_2_O solution (purum p.a., ≥99.0%, Fluka, Seelze, Germany). The suspensions were continuously stirred at ambient temperature for 7 days to promote the incorporation of Zn^2+^ species. After treatment, the solids were separated by filtration, extensively rinsed with distilled water to remove residual electrolyte, and dried in air at 80 °C overnight. Subsequently, the samples were calcined in air at 500 °C for 5 h to stabilize the incorporated zinc species and remove any residual nitrates. Energy-dispersive X-ray spectroscopy (EDS) analysis indicated similar zinc loadings for both materials, reaching 1.2 ± 0.2 wt. % for the Y zeolite and 1.2 ± 0.4 wt. % for the ZSM5 zeolite.

### 4.2. PEO Experimental Conditions

Rectangular samples of AA1050 aluminum alloy (Goodfellow, Huntington, UK) were set as the anode and used as the support for zeolite immobilization. A stainless steel sheet with an area of approximately 20 cm^2^ was used as the cathode in all experiments. The anode material was sealed with insulation resin leaving an active surface area of roughly 3 cm^2^ accessible to electrolyte. Aqueous solution of 10 g/L Na_2_SiO_3_ + 1 g/L KOH was used as the supporting electrolyte for all experiments, with additions of 4 g/L of ZSM5, ZSM5 exchanged with Zn, Y, and Y exchanged with Zn zeolites, as well as 2 g/L ZnO and 4 g/L of a zeolite with 2 g/L ZnO. The electrolyte was prepared using double-distilled and deionized water and p.a. (pro analysis)-grade chemical compounds. The PEO process was carried out in a jacketed electrolytic cell maintaining the temperature of the electrolyte below 30 °C. During PEO processing, all electrolytes with zeolite addition were agitated by a magnetic stirrer. A custom-built power supply (PEO-LAB, Manchester, UK) working in galvanostatic (current-controlled) mode was used for this experiment. The utilized power supply operates in both unipolar and bipolar modes. As the aim of this study was to compare the effects of unipolar and bipolar processing modes, the selected electrical parameters were intended to provide comparable energy input in both modes; therefore, the duty cycle D_t_ of 50% in the unipolar mode, i.e., anodic part of the bipolar mode, was selected. A constant current density of 150 mA/cm^2^ was chosen for the unipolar mode, while that was the value of the anodic current density in bipolar mode. The cathodic vs. anodic current density ratio was R = 1.3.

### 4.3. Characterization Methods

Electrical conductivity of the utilized electrolytes was measured before and after PEO processing using a conductivity meter (Adwa, AD32, Szeged, Hungary), while their pH values were measured with a pH meter (Adwa, AD12).

A scanning electron microscope (SEM, Tescan Vega3 SB, Brno, Czech Republic) in BSE UniVac mode was used to examine the surface morphology of the PEO layers. The chemical composition of the coatings’ top surface was analyzed with an energy dispersive spectrometer (EDS, eumeX IXRFsystems, Heidenrod, Germany) with 120 s integration time and 15 kV acceleration voltage, on a 40 × 50 µm area measurement. The presented EDS results are averaged from five different surface areas. In the case of the oxide coatings obtained by PEO, it is suggested that accuracy of ± 5% should be used [66], and ±5% for O (given as standard deviation by the instrument). For cross-sectional SEM analyses, samples were embedded in epoxy resin and polished with 220, 1000, and 4000 SiC abrasive papers, followed by polishing with 1 μm diamond paste. Cross-sectional micrographs were acquired using JEOL 840A (JEOL, Tokyo, Japan) with an SE detector.

Porosity and surface roughness were estimated using ImageJ software (version: 1.54g, Wayne Rasband and contributors National Institutes of Health, Bethesda, MD, USA). For porosity analysis, SEM images were first converted to 8-bit grayscale, followed by application of a global threshold to distinguish pores from the surrounding matrix based on intensity differences. The threshold was adjusted to ensure accurate segmentation of pore regions and subsequently used to generate binary images.

Surface roughness was evaluated separately using the SurfCharJ 1q plugin of the ImageJ software. In this case, images were converted to 32-bit format, and roughness parameters were calculated from the grayscale intensity distribution.

For each sample, five SEM images acquired from different surface regions were analyzed to account for local morphological variations.

A Rigaku Ultima IV diffractometer (Tokyo, Japan) with an Ni-filtered CuKα radiation source was used for crystal phase identification. Crystallographic data was collected in Bragg–Brentano mode, in 2θ range from 20° to 80° with a scanning rate of 2°/min.

Photoluminescence (PL) spectral measurements were taken on a Horiba Jobin Yvon Fluorolog FL3–22 spectrofluorometer (Piscataway, NJ, USA) at room temperature, with a 450 W xenon lamp (Ushio, Oude Meer, The Netherlands) as the excitation light source. Obtained spectra were corrected for the spectral response of the measuring system and spectral distribution of excitation light source. The excitation wavelength was set to 285 nm, and a cut-off filter was used to avoid the second-order reflections.

Photocatalytic activity of the obtained coatings on the Al substrate was determined by degrading methyl orange (MO) at room temperature. Samples were immersed into 10 mL of 8 mg/L aqueous MO solution and placed on a perforated holder with a magnetic stirrer underneath. In order to attain adsorption–desorption equilibrium, the catalyst and the solutions were stirred in the dark for 30 min. For irradiation, an Osram Vitalux lamp (300 W) (Regensburg, Germany) with irradiation intensity of 16,000 lx that simulates the solar spectrum was placed 25 cm above the top surface of the solution. A fixed quantity of the MO solution (1 mL) was removed every hour to measure the absorption and then to determine concentration using a UV-Vis spectrophotometer Agilent Cary 60 (Santa Clara, CA, USA). After each measurement, the probe solution was returned back to the photocatalytic reactor. Prior to photocatalysis measurements, MO solution was tested for photolysis in the absence of the photocatalyst in order to examine its stability. The lack of change in the MO concentration after 6 h of irradiation revealed that degradation was only due to the presence of the photocatalyst. The presented photocatalysis results are averaged results of four measurements (two samples tested on both sides). The photocatalytic results represent the overall performance of coatings with identical geometric area; normalization to active mass or real surface area was not performed, as the study aims to evaluate functional coatings as integrated systems [10,11,19,20,27,56].

## 5. Conclusions

This study systematically investigated the influence of unipolar and bipolar PEO electrical modes on the incorporation of pure and Zn-exchanged zeolites (ZSM5 and Y) into aluminum oxide coatings under comparable energy input conditions, with particular emphasis on the role of ZnO added individually or in combination with zeolites. The results showed that unipolar PEO produced coatings with higher surface porosity, more developed morphology, and greater incorporation of Zn-containing species, leading to enhanced photocatalytic activity with methyl orange degradation efficiencies of up to 45% after 6 h of irradiation. Coatings formed in electrolytes containing both ZnO and zeolites exhibited higher Zn concentrations and stronger photoluminescence responses than those containing only ZnO, indicating improved charge carrier behavior and confirming that zeolites act as effective carriers for Zn species during the PEO process. Among the investigated zeolites, zeolite Y provided superior Zn incorporation compared to ZSM5 due to its larger pore size, higher ion-exchange capacity, and greater accessibility of active sites. The demonstrated synergy between ZnO and zeolitic carriers opens new possibilities for optimizing PEO-derived photocatalytic surfaces through controlled additive engineering and process parameter selection.

## Figures and Tables

**Table 1:** Samples nomenclature.

Sample	Electrolyte	pH	Conductivity [mS]
SE	10 g/L Na_2_SiO_3_ + 1 g/L KOH	12.13	12.95
SE + ZnO	10 g/L Na_2_SiO_3_ + 1 g/L KOH + 2 g/L ZnO	12.24	13.03
SE + ZSM5	10 g/L Na_2_SiO_3_ + 1 g/L KOH + 4 g/L ZSM5	12.21	13.28
SE + Zn-ZSM5	10 g/L Na_2_SiO_3_ + 1 g/L KOH + 4 g/L Zn-ZSM5 (Zn exchanged)	12.42	13.44
SE + ZSM5 + ZnO	10 g/L Na_2_SiO_3_ + 1 g/L KOH + 4 g/L ZSM5 + 2 g/L ZnO	12.31	14.01
SE + Y	10 g/L Na_2_SiO_3_ + 1 g/L KOH + 4 g/L Y	12.71	13.65
SE + Zn-Y	10 g/L Na_2_SiO_3_ + 1 g/L KOH + 4 g/L Zn-Y (Zn exchanged)	12.37	14.21
SE + Y + ZnO	10 g/L Na_2_SiO_3_ + 1 g/L KOH + 4 g/L Y + 2 g/L ZnO	12.42	14.42

**Table 2:** Thickness of the specimens treated in unipolar and bipolar processing mode.

Sample	Thickness [µm]	
	Unipolar	Bipolar
SE	5.62 ± 0.13	10.94 ± 0.11
SE + ZnO	6.88 ± 0.11	11.25 ± 0.11
SE + ZSM5	10.33 ± 0.19	8.44 ± 0.12
SE + Zn-ZSM5	8.12 ± 0.15	8.92 ± 0.09
SE + ZSM5 + ZnO	14.08 ± 0.11	9.34 ± 0.16
SE + Y	7.82 ± 0.12	7.50 ± 0.14
SE + Zn-Y	9.68 ± 0.16	8.46 ± 0.15
SE + Y + ZnO	14.21 ± 0.14	9.29 ± 0.11

**Table 3:** Porosity and roughness of specimens treated in unipolar and bipolar processing mode.

Sample	Porosity [%]		Roughness [µm]	
	Unipolar	Bipolar	Unipolar	Bipolar
SE	8.25 ± 0.19	4.33 ± 0.11	26.27 ± 0.74	27.09 ± 0.91
SE + ZnO	10.71 ± 0.23	7.30 ± 0.09	24.84 ± 0.81	22.60 ± 0.91
SE + ZSM5	7.02 ± 0.16	6.37 ± 0.12	18.05 ± 0.72	22.09 ± 0.84
SE + Zn-ZSM5	11.74 ± 0.25	5.60 ± 0.12	22.32 ± 0.74	20.69 ± 0.87
SE + ZSM5 + ZnO	13.13 ± 0.28	6.50 ± 0.12	17.91 ± 0.82	23.37 ± 0.85
SE + Y	7.28 ± 0.18	6.09 ± 0.11	17.32 ± 0.85	21.95 ± 0.79
SE + Zn-Y	11.42 ± 0.21	5.18 ± 0.08	18.77 ± 0.79	21.34 ± 0.81
SE + Y + ZnO	12.94 ± 0.21	6.13 ± 0.09	23.81 ± 0.81	19.63 ± 0.78

**Table 4:** EDS data for all the samples treated in unipolar PEO processing mode.

Element	Al	O	Na	Si	K	Zn
Sample	Weight Percent [wt. %]					
SE	47.7	46.7	1.4	3.4	0.8	N/A
SE + ZnO	47.8	44.1	0.9	4.7	0.2	2.3
SE + ZSM5	47.1	46.6	2.2	3.9	0.2	N/A
SE + Zn-ZSM5	47.4	43.8	1.6	4.9	0.2	2.1
SE + ZSM5 + ZnO	46.4	42.2	1.8	5.1	0.3	4.2
SE + Y	46.2	43.6	1.7	8.0	0.5	N/A
SE + Zn-Y	46.2	43.3	2.0	5.7	0.2	2.6
SE + Y + ZnO	46.3	42.5	1.3	5.2	0.1	4.6

**Table 5:** EDS data for all the samples treated in bipolar PEO processing mode.

Element	Al	O	Na	Si	K	Zn
Sample	Weight Percent [wt. %]					
SE	54.3	40.7	2.7	2.2	0.1	N/A
SE + ZnO	49.6	41.8	4.4	2.1	0.3	1.8
SE + ZSM5	50.4	42.7	3.7	3.1	0.1	N/A
SE + Zn-ZSM5	49.3	41.5	4.1	3.6	0.2	1.3
SE + ZSM5 + ZnO	48.0	42.2	4.2	3.3	0.2	2.1
SE + Y	50.6	40.1	4.4	5.1	0.1	N/A
SE + Zn-Y	49.7	39.7	3.9	4.9	0.2	1.6
SE + Y + ZnO	48.4	41.3	3.3	4.5	0.1	2.4

**Table 6:** Pseudo-first-order *k_app_* values for all obtained PEO coatings.

Sample	*k_app_* [min^−1^]	
	Unipolar	Bipolar
SE	0.0031	0.0012
SE + ZnO	0.0054	0.0041
SE + ZSM5	0.0038	0.0021
SE + Zn-ZSM5	0.0053	0.0026
SE + ZSM5 + ZnO	0.0072	0.0047
SE + Y	0.0034	0.0023
SE + Zn-Y	0.0048	0.0028
SE + Y+ZnO	0.0075	0.0051

## Data Availability

The raw data supporting the conclusions of this article will be made available by the authors on request.

## Abbreviations

The following abbreviations are used in this manuscript:

PEO	Plasma Electrolytic Oxidation
SEM	Scanning Electron Microscope
XRD	X-ray Diffraction
PL	Photoluminescence
MO	Methyl Orange

## References

[1] Das A., Sahoo R.K., Mishra D.K., Singh S.K., Mane R.S., Kim K.H. (2019). Thermal plasma- inspired synthesis of ZnO_1−x_Mn_x_ dilute magnetic semiconductors for enhanced visible light photocatalysis. Appl. Surf. Sci..

[2] Temerov F., Pham K., Juuti P., Makela J.M., Grachova E.V., Kumar S., Eslava S., Saarinen J.J. (2020). Silver-decorated TiO_2_ inverse opal structure for visible light-induced photocatalytic degradation of organic pollutants and hydrogen evolution. ACS Appl. Mater. Interfaces.

[3] Moreira N.F.F., Sampaio M.J., Ribeiro A.R., Silva C.G., Faria J.L., Silva A.M.T. (2019). Metal-free g-C_3_N_4_ photocatalysis of organic micropollutants in urban wastewater under visible light. Appl. Catal. B Environ..

[4] Kanakkillam S., Shaji S., Krishnan B., Vazquez-Rodriguez S., Martinez J.A.A., Palma M.I.M., Avellaneda D.A. (2020). Nanoflakes of zinc oxide:cobalt oxide nanocomposites by pulsed laser fragmentation for visible light photocatalysis. Appl. Surf. Sci..

[5] Ighadon A.O., Fitzpatrick P. (2013). Heterogeneous photocatalysis: Recent advances and applications. Catalysts.

[6] Samhaber W.M., Nguyen M.T. (2014). Applicability and costs of nanofiltration in combination with photocatalysis for the treatment of dye house effluents. Beilstein J. Nanotechnol..

[7] Lu X., Blawert C., Zheludkevich M.L., Kainer K.U. (2015). Insights into plasma electrolytic oxidation treatment with particle addition. Corros. Sci..

[8] Lu X., Blawert C., Huang Y., Ovri H., Zheludkevich M.L., Kainer K.U. (2016). Plasma electrolytic oxidation coatings on Mg alloy with addition of SiO_2_ particles. Electrochim. Acta.

[9] Liu W., Pu Y., Liao H., Lin Y., He W. (2020). Corrosion and wear behavior of PEO coatings on D16T aluminum alloy with different concentrations of graphene. Coatings.

[10] Mojsilović K., Božović N., Stojanović S., Damjanović-Vasilić L., Serdechnova M., Blawert C., Zheludkevich M.L., Vasilić R., Stojadinović S. (2021). Zeolite-containing photocatalysts immobilized on aluminum support by plasma electrolytic oxidation. Surf. Interfaces.

[11] Mojsilović K., Lačnjevac U., Stojanović S., Damjanović-Vasilić L., Vasilić R., Stojadinović S. (2021). Formation and properties of oxide coatings with immobilized zeolites obtained by plasma electrolytic oxidation of aluminum. Metals.

[12] Yerokhin A.L., Nie X., Matthews A., Dowey S.J. (1999). Plasma electrolysis for surface engineering. Surf. Coat. Technol..

[13] Kaseem M., Fatimah S., Nashrah N., Ko Y.G. (2021). Recent progress in surface modification of metals coated by plasma electrolytic oxidation: Principle, structure, and performance. Prog. Mater. Sci..

[14] Clyne T.W., Troughton S.C. (2019). A review of recent work on discharge characteristics during plasma electrolytic oxidation of various metals. Int. Mater. Rev..

[15] Pezzato L., Kostelac L., Tonelli L., Elsayed H., Kajánek D., Bernardo E., Martini C., Dabalà M., Brunelli K. (2025). Effect of different types of glass powders on the corrosion and Wear resistance of PEO coatings produced on 6061 aluminum alloy. Met. Mater. Int..

[16] Grebnevs V., Dulski M., Husak Y., Bertins M., Reinholds I., Babilas D., Iatsunskyi I., Coy E., Kazek-Kęsik A., Rawicka P. (2025). Advancements in plasma electrolytic oxidation with particle suspensions: A novel approach for the direct incorporation of calcium carbonate. Appl. Surf. Sci..

[17] Jiang C., Wang Y., Chen Q., Ying G., Fei Q. (2025). Unveiling the role of SiC particle reinforcement on aluminum matrix composites surface: Insights into PEO coating growth, electrical insulation, and corrosion resistance. Ceram. Int..

[18] Ceriani F., Casanova L., Massimini L., Brenna A., Ormellese M. (2023). TiO_2_ microparticles incorporation in coatings produced by plasma electrolytic oxidation (PEO) on titanium. Coatings.

[19] Mojsilović K., Serdechnova M., Blawert C., Zheludkevich M.L., Stojadinović S., Vasilić R. (2024). In-situ incorporation of LDH particles during PEO processing of aluminium alloy AA2024. Appl. Surf. Sci..

[20] Kasneryk V., Wu T., Rohr H., Serdechnova M., Mojsilović K., Wieland D.C.F., Davydok A., Gazenbiller E., Vasilić R., Blawert C. (2024). Controllable recrystallization of ZnO/ZnAl_2_O_4_ based PEO into ZIF-8 as a route for the formation of multifunctional coatings. J. Ind. Eng. Chem..

[21] Burton A.W., Chester A.W., Derouane E.G. (2009). Powder Diffraction in Zeolite Science. Zeolite Characterization and Catalysis: A Tutorial.

[22] Alvarez-Aquinaga E.A., Elizade-Gonzalez M.P., Sabinas-Hernandez S.A. (2020). Unpredicted photocatalytic activity of clinoptilolite-morderite natural zeolite. RSC Adv..

[23] Latha P., Karuthapandian S. (2017). Novel, facile and swift technique for synthesis of CeO_2_ nanocubes immobilized on zeolite for removal of CR and MO dye. J. Clust. Sci..

[24] Dias S.A.S., Marques A., Lamaka S.V., Simoes A., Diamantino T.C., Ferreira M.G.S. (2013). The role of Ce(III)-enriched zeolites on the corrosion protection of AA2024-T3. Electrochim. Acta.

[25] Al Abri S., Rogov A., Aliasghari S., Bendo A., Matthews A., Yerokhin A., Mingo B. (2024). In-situ incorporation of Ce-zeolite during soft sparking plasma electrolytic oxidation. J. Mater. Res. Technol..

[26] Al Abri S., Knowles T., Pan Y., Yerokhin A., Mingo B. (2025). Multilayer PEO coatings with encapsulated cerium for active corrosion protection of aluminium. npj Mater. Degrad..

[27] Stojadinović S., Radić N., Tadić N., Vasilić R., Grbić B. (2020). Enhanced ultraviolet light driven photocatalytic activity of ZnO particles incorporated by plasma electrolytic oxidation into Al_2_O_3_ coatings co-doped with Ce^3+^. Opt. Mater..

[28] Yu Y., Du A., Wang C., Ma R., Fan Y., Zhao X., Cao X. (2021). Incorporation mechanism of ZnO nanoparticles in PEO coating on 1060 Al alloy. Surf. Coat. Technol..

[29] Huang Q., Wu Z., Wu H., Ji S., Ma Z., Wu Z., Chen P., Zhu J., Fu R.K.Y., Lin H. (2019). Corrosion behavior of ZnO-reinforced coating on aluminum alloy prepared by plasma electrolytic oxidation. Surf. Coat. Technol..

[30] Lu X., Mohedano M., Blawert C., Matykina E., Arrabal R., Kainer K.U., Zheludkevich M.L. (2016). Plasma electrolytic oxidation coatings with particle additions—A review. Surf. Coat. Technol..

[31] Mojsilović K., Blawert C., Serdechnova M., Zheludkevich M.L. (2026). Thermal conductivity of PEO-coated AlMg3 samples with different particle additions. Int. Commun. Heat Mass Transf..

[32] Mojsilović K., Serdechnova M., Blawert C., Kasneryk V., Zhang Z., Wieland D.C.F., Vasilić R., Zheludkevich M.L. (2025). Tailoring plasma electrolytic oxidation through metallic cation addition: Insights from bipolar and unipolar electrical regimes. Appl. Surf. Sci. Adv..

[33] Arrabal R., Matykina E., Hashimoto T., Skeldon P., Thompson G.E. (2009). Characterization of AC PEO coatings on magnesium alloys. Surf. Coat. Technol..

[34] Cheng Y., Zhang Q., Zhu Z., Tu W., Cheng Y., Skeldon P. (2010). Potential and morphological transitions during bipolar plasma electrolytic oxidation of tantalum in silicate electrolyte. Ceram. Int..

[35] Rogov A.B., Nemcova A., Hashimoto T., Matthews A., Yerokhin A. (2022). Analysis of electrical response, gas evolution and coating morphology during transition to soft sparking PEO of Al. Surf. Coat. Technol..

[36] Hakimizad A., Raeissi K., Santamaria M., Asghari M. (2018). Effects of pulse current mode on plasma electrolytic oxidation of 7075 Al in Na_2_WO_4_ containing solution: From unipolar to soft-sparking regime. Electrochim. Acta.

[37] Tang Q., Qiu T., Ni P., Zhai D., Shen J. (2022). Soft sparking discharge mechanism of micro-arc oxidation occurring on titanium alloys in different electrolytes. Coatings.

[38] Cheng Y., Feng T., Cheng Y. (2022). A systematic study of the role of cathodic polarization and new findings on the soft sparking phenomenon from plasma electrolytic oxidation of an Al-cu-Li alloy. Electrochem. Soc..

[39] Karlova P., Serdechnova M., Blawert C., Lu X., Mohedano M., Tolnai D., Zeller-Plumhoff B., Zheludkevich M.L. (2022). Comparison of 2D and 3D Plasma Electrolytic Oxidation (PEO)-Based Coating Porosity Data Obtained by X-ray Tomography Rendering and a Classical Metallographic Approach. Materials.

[40] Nominé A., Martin J., Henrion G., Belmonte T. (2015). Effect of cathodic micro-discharges on oxide growth during plasma electrolytic oxidation (PEO). Surf. Coat. Technol..

[41] Jaspard-Mécuson F., Czerwiec T., Henrion G., Belmonte T., Dujardin L., Viola A., Beauvir J. (2007). Tailored aluminium oxide layers by bipolar current adjustment in the plasma electrolytic oxidation (PEO) process. Surf. Coat. Technol..

[42] Jiang C., Wang Y., Wang S., Li Y., Zou Y., Ouyang J., Jia D., Zhou Y. (2022). Achieving high-efficiency electrically insulating ceramic layer formed on SiCp/Al composite by bipolar pulsed PEO for novel integrated strategy. Surf. Coat. Technol..

[43] Qian C., Chen B., Li H., Shi R., Yang Z., Zhang N., Song S., Liu C., Yang B. (2023). Growth behavior and Wear resistance of PEO coatings in bipolar mode. Trans. Indian Inst. Met..

[44] Ruggiu A., Carvalho A.P., Rombi E., Martins A., Rocha J., Parpot P., Neves I.C., Cutrufello M.G. (2024). Y and ZSM-5 Hierarchical Zeolites Prepared Using a Surfactant-Mediated Strategy: Effect of the Treatment Conditions. Materials.

[45] Algieri C., Drioli E., Donato L. (2013). Development of mixed matrix membranes for controlled release of ibuprofen. J. Appl. Polym. Sci..

[46] Putra A.T.S.P. (2020). An improved method for high photocatalytic performance of ZnAl_2_O_4_ spinel derived from layered double hydroxide precursor. SN Appl. Sci..

[47] Shah S.A.A., Gkoulemani N., Irvine J.T.S., Sajjad M.T., Baker R.T. (2024). Synthesis of high surface area mesoporous ZnAl2O4 with excellent photocatalytic activity for the photodegradation of Congo Red dye. J. Catal..

[48] Chang J.S., Strunk J., Chong M.N., Poh P.E., Ocon J.D. (2020). Multi-dimensional zinc oxide (ZnO) nanoarchitectures as efficient photocatalysts: What is the fundamental factor that determines photoactivity in ZnO?. J. Hazard. Mater..

[49] Stojadinović S., Vasilić R., Nedić Z., Kasalica B., Belča I., Zeković L. (2011). Photoluminescent properties of barrier anodic oxide films on aluminum. Thin Solid Films.

[50] Li P.G., Lei M., Tang W.H. (2010). Raman and photoluminescence properties of α-Al2O3 microcones with hierarchical and repetitive superstructure. Mater. Lett..

[51] Panigrahi S.K., Mishra A.K. (2019). Inner filter effect in fluorescence spectroscopy: As a problem and as a solution. J. Photochem. Photobiol. C Photochem. Rev..

[52] Benkhaya S., M’rabet S., El Harfi A. (2020). Classifications, properties, recent synthesis and applications of azo dyes. Heliyon.

[53] Yan H., Hou J., Fu Z., Yang B., Yanh P., Liu K., Wen M., Chen Y., Fu S., Li F. (2009). Growth and photocatalytic properties of one-dimensional ZnO nanostructures prepared by thermal evaporation. Mater. Res. Bull..

[54] González M.D., Cesteros Y., Salagre P. (2011). Comparison of dealumination of zeolites beta, mordenite and ZSM-5 by treatment with acid under microwave irradiation. Microporous Mesoporous Mater..

[55] Liu H., Wang Y., Yuan H. (2025). Hierarchical Meso-Microporous ZSM-5 Zeolite for Producing Light Olefins by Catalytically Cracking Oleic Acid. Energy Fuels.

[56] Stojadinović S., Tadić N., Radić N., Stojadinović B., Grbić B., Vasilić R. (2015). Synthesis and characterization of Al_2_O_3_/ZnO coatings formed by plasma electrolytic oxidation. Surf. Coat. Technol..

[57] Wang T., Wu H., Chen C., Liu C. (2012). Growth, optical, and electrical properties ofnonpolar M-plane ZnO on p-Si substrates with Al_2_O_3_ buffer layers. Appl. Phys. Lett..

[58] Giziński D., Mojsilović K., Brudzisz A., Tiringer U., Vasilić R., Taheri P., Stępniowski W.J. (2022). Controlling the Morphology of Barrel-Shaped Nanostructures Grown via CuZn Electro-Oxidation. Materials.

[59] Razali M., Dris M.R.M., Rudin N.N.S.M. (2009). Photodegradation of methyl orange dye using titanium dioxide photocatalyst. J. Sustain. Sci. Manag..

[60] Khalifa Z.S., Shaban M., Ahmed I.A. (2023). Photocatalytic Degradation of Methyl Orange and Methylene Blue Dyes by Engineering the Surface Nano-Textures of TiO_2_ Thin Films Deposited at Different Temperatures via MOCVD. Molecules.

[61] Bani-Atta S.A., Darwish A.A.A., Shwashreh L., Alotaibi F.A., Al-Tweher J.N., Al-Aoh H.A., El-Zaidia E.F.M. (2024). Efficient Photocatalytic Degradation of Methylene Blue and Methyl Orange Using Calcium-Polyoxometalate Under Ultraviolet Irradiation. Processes.

[62] Aljuaid A., Almehmadi M., Alsaiari A.A., Allahyani M., Abdulaziz O., Alsharif A., Alsaiari J.A., Saih M., Alotaibi R.T., Khan I. (2023). g-C_3_N_4_ Based Photocatalyst for the Efficient Photodegradation of Toxic Methyl Orange Dye: Recent Modifications and Future Perspectives. Molecules.

[63] Friedemann A.E.R., Thiel K., Gesing T.M., Plagemann P. (2018). Photocatalytic activity of TiO2 layers produced with plasma electrolytic oxidation. Surf. Coat. Technol..

[64] Manojkumar P., Lokeshkumar E., Saikiran A., Govardhanan B., Ashok M., Rameshbabu N. (2020). Visible light photocatalytic activity of metal (Mo/V/W) doped porous TiO_2_ coating fabricated on Cp-Ti by plasma electrolytic oxidation. J. Alloys Compd..

[65] Chaharmahali R., Fattah-alhosseini A., Karbasi M. (2025). Unveiling enhanced photocatalytic behavior: Plasma electrolytic oxidation for TiO_2_/Bi_2_WO_6_ heterojunction coatings to enhance the photocatalytic degradation of methylene blue under visible light. Ceram. Int..

[66] Hussein R.O., Nie X., Northwood D.O., Yerokhin A., Matthews A. (2010). Spectroscopic study of electrolytic plasma and discharging behaviour during the plasma electrolytic oxidation (PEO) process. J. Phys. D Appl. Phys..

